# Outcomes of a Physiology-driven Extracardiac Fontan Strategy Incorporating Computational Fluid Dynamics: A Multicentre Study

**DOI:** 10.1093/ejcts/ezag145

**Published:** 2026-04-03

**Authors:** Onur Benli, Mustafa Kemal Avşar, Ahmet Şen, İbrahim Özgür Önsel, Barış Kırat, Cenap Zeybek

**Affiliations:** Department of Cardiovascular Surgery, Cukurova University Faculty of Medicine, Adana, 01330, Turkey; Department of Cardiovascular Surgery, Cukurova University Faculty of Medicine, Adana, 01330, Turkey; Department of Cardiovascular Surgery, Cukurova University Faculty of Medicine, Adana, 01330, Turkey; Department of Anesthesiology and Reanimation, Medicana International Istanbul Hospital, Istanbul, 34520, Turkey; Department of Anesthesiology and Reanimation, Medicana International Istanbul Hospital, Istanbul, 34520, Turkey; Department of Pediatric Cardiology, Medipol University, Istanbul, 34810, Turkey

**Keywords:** Fontan procedure, extracardiac total cavopulmonary connection, era-based management, single-ventricle physiology, long-term outcomes

## Abstract

**Objectives:**

To report early and mid-term outcomes after extracardiac total cavopulmonary connection (EC-TCPC) and to evaluate prespecified subgroup comparisons, including calendar-time era effects (Era I vs Era II), selective fenestration, conduit-size categories, and late-era computational fluid dynamics (CFD)-guided conduit sizing.

**Methods:**

This multicentre retrospective cohort study included 788 patients who underwent EC-TCPC between 2009 and 2025. Patients were prespecified into Era I (2009-2016) and Era II (2017-2025) for calendar-time comparisons reflecting evolving pathway-based management; the CFD-guided subcohort was restricted to late-era patients. Survival and event-related outcomes were analysed using Kaplan-Meier, competing-risk methods, and multivariable regression models.

**Results:**

Early mortality was 2.4%. During a median follow-up of 8.2 years, overall survival was 97.2% at 1 year, 95.0% at 5 years, and 92.5% at 10 years. Freedom from major adverse events (MAEs) was 95.6%, 84.3%, and 75.3% at the same time points; the 10-year cumulative incidence of thromboembolism was 6.8% and of MAEs was 23.6%. In the prespecified era comparison, Era II was associated with shorter pleural drainage duration and lower rates of prolonged pleural effusion. Fenestrated patients showed higher event rates, consistent with confounding by indication. In Era II, CFD-guided conduit sizing was associated with improved early postoperative haemodynamics but did not independently reduce mid-term clinical events after adjustment.

**Conclusions:**

A physiology-driven extracardiac Fontan strategy provides low early mortality and preserved mid-term survival. Selective fenestration and geometry-aware conduit sizing support individualized surgical planning, whereas CFD-guided optimization should be regarded as a decision-support tool rather than a causal determinant of outcome.

## Graphical abstract

**Figure:**
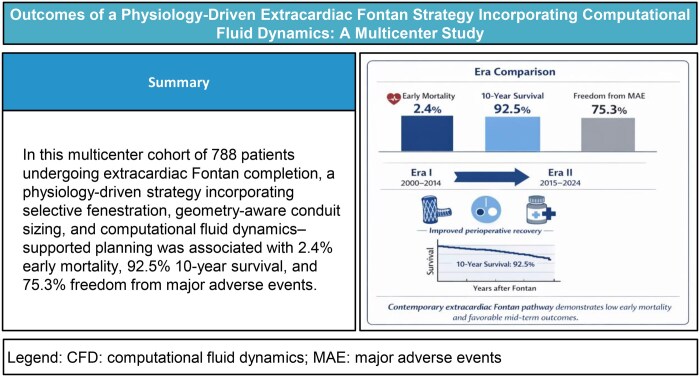

## Introduction

The Fontan operation has substantially improved survival in single-ventricle physiology, and extracardiac total cavopulmonary connection (EC-TCPC) is widely used in contemporary practice, with many cohorts reporting 10-year survival in the low-90% range.^1–5^ Despite these advances, Fontan circulation remains physiologically fragile and highly sensitive to venous hypertension and changes in pulmonary vascular resistance and ventricular compliance.^6^^,^^7^

Conduit sizing is a key determinant of cavopulmonary energetics. Patient-specific computational fluid dynamics (CFD) can quantify power loss (PL) and flow distribution, supporting geometry-aware conduit selection beyond body-size-based approaches.^8^^,^^9^ However, in real-world multicentre practice—where perioperative pathways, fenestration policies, and surveillance strategies evolve over time—the incremental clinical impact attributable to CFD-guided planning remains insufficiently defined.^10^

Therefore, we report a 4-centre cohort of 788 consecutive extracardiac Fontan completions (2009-2025) to describe early and mid-term outcomes, evaluate era-related changes in management, and assess whether CFD-guided conduit sizing is associated with improved postoperative haemodynamics and event-related outcomes after adjustment for clinical risk. Beyond reporting outcomes from a large multicentre EC-TCPC cohort in an under-represented region, this study adds: (i) transparent cohort assembly and follow-up ascertainment, (ii) adjudicated non-fatal end-points reported with competing-risk methods (death as competing event), and (iii) a real-world evaluation of patient-specific CFD as a decision-support adjunct within an evolving physiology-driven pathway, without causal attribution.

## Methods

### Study design and population

This retrospective multicentre cohort study included 788 consecutive patients with functionally univentricular heart disease who underwent EC-TCPC between 2009 and 2025 at 4 tertiary centres in Türkiye and Iraq (**Table S5**). Each centre maintains a prospectively curated congenital cardiac surgery registry. De-identified data were extracted using a standardized data dictionary and verified against electronic medical records. A consortium guideline established in 2010 harmonized candidacy assessment, conduit configuration/sizing, selective fenestration criteria, and antithrombotic strategy. The study was approved by the coordinating ethics committee (Medicana IRB-2025/112) and complied with the Declaration of Helsinki and the Declaration of Taipei; informed consent was waived due to the retrospective use of de-identified data.

The consortium pathway comprised 3 components: (i) physiology-anchored pre-Fontan assessment (haemodynamics, ventricular function, atrioventricular valve regurgitation, and pulmonary artery morphology), (ii) intraoperative configuration decisions including geometry-aware conduit sizing (with patient-specific CFD decision support in a late-era subcohort) and selective fenestration guided by immediate post-bypass haemodynamics/perfusion, and (iii) a standardized perioperative management pathway emphasising low airway pressures, early negative fluid balance, modified ultrafiltration, and a protocolized pleural effusion/chylothorax escalation strategy.

**Figure 1.**
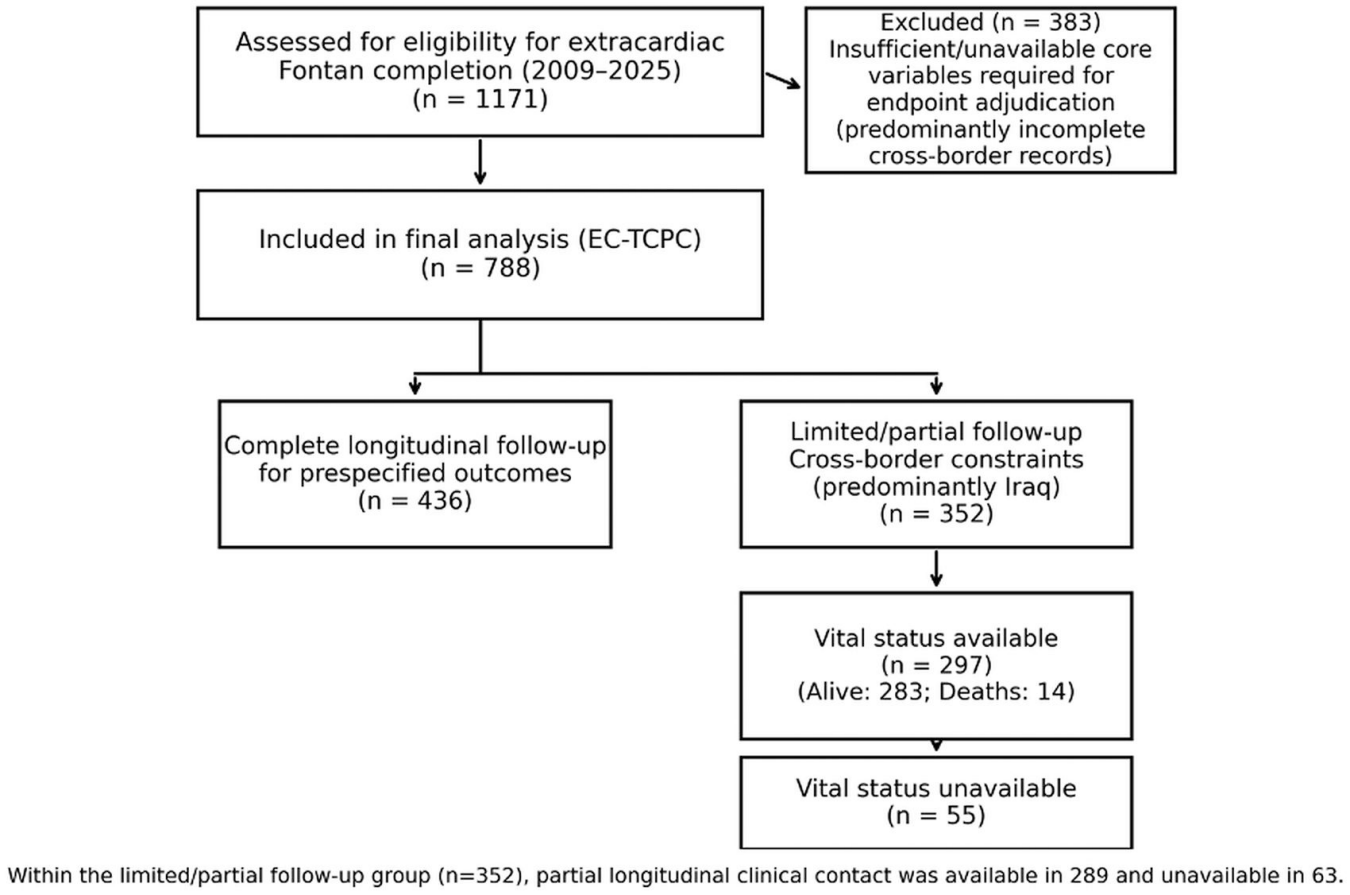
Cohort Assembly and Follow-up Ascertainment. Of 1171 patients assessed for eligibility (2009-2025), 383 were excluded due to insufficient/unavailable core variables required for end-point adjudication, leaving 788 EC-TCPC patients for the final analysis. Complete longitudinal follow-up for prespecified outcomes was available in 436 patients; 352 had limited follow-up primarily due to cross-border follow-up constraints (predominantly cases operated in Iraq). Within the limited follow-up group, vital status was available for 297 patients (283 alive; 14 deaths) and unavailable for 55. Cause of death could not be reliably ascertained for a subset due to lack of a unified mortality registry. Abbreviation: EC-TCPC, extracardiac total cavopulmonary connection.

Eligible patients had completed staged palliation culminating in EC-TCPC for single-ventricle physiology. Exclusion criteria were insufficient data for end-point adjudication (>20% missing core variables), absence of Fontan completion, major non-cardiac comorbidities expected to confound outcomes, or Fontan takedown/conversion/transplantation during the index admission. Of 1171 patients assessed, 383 were excluded (predominantly incomplete cross-border records), leaving 788 for the final analysis (**Figure 1**). Between 2009 and 2025, 1171 patients were assessed for eligibility for extracardiac Fontan completion across participating centres. Of these, 383 were excluded due to insufficient/unavailable core variables required for end-point adjudication (predominantly incomplete records from cases operated in Iraq), leaving 788 patients for the final analysis (**Figure 1**).

### Data collection and follow-up

A standardized electronic case-report form captured demographic, anatomic, haemodynamic, operative, early postoperative, and longitudinal outcome data using a common data dictionary. At each site, a 10% random sample of cases per quarter underwent structured source verification by local investigators against the electronic medical record using a predefined variable list. Discrepancies were reconciled with the coordinating centre, and overall concordance exceeded 95%.

Patients were prespecified into 2 eras: Era I (2009-2016; *n* = 392) and Era II (2017-2025; *n* = 396). Eras were defined a priori by calendar time and are interpreted as proxies for co-evolving consortium practices rather than mutually exclusive procedural groups; therefore, era comparisons are associative and not causal.

Prespecified subgroup analyses included fenestration status, conduit size category (<18 mm; 18-20 mm; >20 mm), and a late-era CFD subcohort (*n* = 217). Follow-up was anchored to the date of EC-TCPC and patients were censored at the last confirmed clinical contact (median 8.2 years [interquartile range, IQR 4.1-12.5; range 0.5-16.3]). Vital status ascertainment was available for 733/788 patients (93.0%).

Follow-up was anchored to the date of EC-TCPC and patients were censored at the last confirmed clinical contact (median 8.2 years [IQR 4.1-12.5; range 0.5-16.3]). Vital status ascertainment was available for 733/788 patients (93.0%). Complete longitudinal follow-up sufficient for prespecified non-fatal outcome adjudication was available in 436 patients, whereas 352 had limited/partial follow-up due to cross-border constraints (predominantly cases operated in Iraq); within this limited/partial subgroup, vital status was available in 297 (283 alive; 14 deaths) and unavailable in 55, and partial longitudinal clinical contact for non-fatal outcome ascertainment was available in 289 and unavailable in 63.

For time-to-event analyses, patients were censored at last confirmed contact; outcomes were not imputed, whereas missing covariates were handled as described in the Statistical Analysis section.

### End-points and definitions

Primary end-points were early mortality (≤30 days or during index hospitalization), late mortality (>30 days), and major adverse events (MAEs), defined as thromboembolism, protein-losing enteropathy (PLE), clinically significant Fontan-associated liver disease (FALD), or clinically significant arrhythmia requiring intervention.

Clinically significant FALD was prespecified and adjudicated using a standardized surveillance framework across centres. Routine hepatic follow-up included liver biochemistry and abdominal ultrasonography at scheduled visits, with transient elastography performed when available; cross-sectional imaging (contrast-enhanced CT or MRI) and/or endoscopy were obtained when clinically indicated. For end-point adjudication, FALD was classified as clinically significant when at least 1 of the following was present: (i) imaging- or elastography-based evidence of clinically relevant fibrosis/cirrhosis (eg, elastography consistent with ≥F2 fibrosis or cirrhotic morphology/nodularity on imaging), and/or (ii) portal-hypertension-related complications (ascites, varices/variceal bleeding, splenomegaly with thrombocytopaenia, or clinically relevant hepatocellular dysfunction) attributable to Fontan physiology. Cases were adjudicated centrally by the study committee using available longitudinal records and imaging reports; in patients with limited/partial follow-up, FALD outcomes were analysed using available data and censored at last confirmed clinical contact (outcomes were not imputed). These definitions and the surveillance approach were aligned with contemporary consensus frameworks and scientific statements for Fontan follow-up and FALD reporting.^5^^,^^11^

Secondary end-points included prolonged pleural effusion (PPE), cumulative pleural drainage, reintervention, and hospital length of stay. Prolonged pleural effusion was defined as pleural drainage >14 days or escalation beyond standard drainage. A composite Fontan failure end-point included Fontan takedown or conversion, listing for or receipt of heart transplantation, plastic bronchitis, PLE, or persistent low cardiac output requiring prolonged inotropic support beyond postoperative day 14 with objective evidence of hypoperfusion.

End-point definitions were aligned with STS/EACTS congenital database terminology. All suspected events were adjudicated centrally by a multidisciplinary committee using prespecified criteria.

### Surgical technique

All procedures were performed via median sternotomy under moderate hypothermic cardiopulmonary bypass with bicaval cannulation. Extracardiac total cavopulmonary connection was constructed using ring-reinforced expanded polytetrafluoroethylene conduits. Conduit diameter selection integrated body surface area, inferior vena cava caliber, pulmonary artery geometry, and energy-efficiency principles informed by haemodynamic modelling.^8^^,^^9^^,^^12^^,^^13^ Extensive pulmonary artery mobilization facilitated tension-free anastomoses.

Fenestration was not performed routinely. When indicated, a 3-5 mm fenestration was created selectively after separation from cardiopulmonary bypass based on a prespecified haemodynamic-perfusion profile rather than routine application. Consortium criteria incorporated standardized post-bypass haemodynamics (mean pulmonary artery pressure, ventricular end-diastolic pressure, and central venous pressure [CVP]) together with systemic perfusion markers (eg, lactate trend, mixed venous saturation/near-infrared spectroscopy where available, urine output and vasoactive requirement) to identify patients at higher risk of early Fontan pressure load and pleural morbidity. Final decisions remained surgeon-directed within the consortium framework. Concomitant procedures performed at Fontan completion were prospectively recorded, including atrioventricular valve procedures, pulmonary artery reconstruction/augmentation, atrioseptectomy, pacemaker implantation/lead procedures, LVOTR/VSD enlargement when required, and DKS/aortic reconstruction. These concomitant interventions are summarized in the operative characteristics (**Table 1**).

**Table 1.** Operative Characteristics

Characteristic	Overall (*n* = 788)	Era I (*n* = 392)	Era II (*n* = 396)	*P* value
Conduit diameter (mm), mean (SD)	18.47 (1.90)	17.8 (2.1)	19.1 (1.7)	<.001
Conduit diameter category, *n* (%)				<.001
• <18 mm	212 (26.9)	142 (36.2)	70 (17.7)	
• 18–20 mm	456 (57.9)	198 (50.5)	258 (65.2)	
• >20 mm	120 (15.2)	52 (13.3)	68 (17.2)	
Fenestration, *n* (%)	178 (22.6)	121 (30.9)	57 (14.1)	<.001
CPB time (min), mean (SD)	128 (29)	131 (30)	125 (28)	.060
Aortic cross-clamp time (min), mean (SD)^a^	41 (12)	42 (13)	40 (11)	.180
Bilateral PA mobilization, *n* (%)	742 (94.2)	362 (92.3)	380 (96.0)	.020
Concomitant procedures at Fontan completion^†^, *n* (%)				
• Any concomitant procedure	307 (39.0)	181 (46.2)	126 (31.8)	<.001
• AVV procedure	108 (13.7)	47 (12.0)	61 (15.4)	.163
• PA reconstruction/plasty	55 (7.0)	30 (7.7)	25 (6.3)	.460
• Atrioseptectomy	95 (12.1)	42 (10.7)	53 (13.4)	.250
• Pacemaker implantation/lead procedure	34 (4.3)	11 (2.8)	23 (5.8)	.038
• LVOTR/VSD enlargement	16 (2.0)	7 (1.8)	9 (2.3)	.628
• DKS/aorta reconstruction	8 (1.0)	4 (1.0)	4 (1.0)	1.000

Values are presented as mean (SD) or *n* (%), as appropriate. *P* values were derived from Student’s *t*-test (continuous variables) and χ^2^/Fisher’s exact test (categorical variables). Aortic cross-clamp time is calculated only among patients in whom cross-clamping was performed and is therefore reported on a conditional basis.Concomitant procedures at Fontan completion†: patients may have undergone more than one concomitant procedure; therefore, percentages are not mutually exclusive and may not sum to 100%.

Abbreviations: CPB, cardiopulmonary bypass; ePTFE, expanded polytetrafluoroethylene; PA, pulmonary artery.

a Calculated among patients who required cross-clamping.

#### Computational fluid dynamics

In the final 217 patients, conduit sizing was additionally supported by patient-specific CFD derived from contrast-enhanced computed tomography angiography (CTA). Computational fluid dynamics was used as decision support within the consortium’s standard geometry-aware sizing approach (body surface area, caval dimensions, and branch pulmonary artery geometry) and was integrated with operative judgement and intraoperative haemodynamics rather than used in isolation.

Pre-Fontan CTA datasets (slice thickness 0.5-1.0 mm; in-plane resolution 0.5-0.6 mm) were imported into Materialise Mimics (v21.0). The cavopulmonary pathway (inferior vena cava (IVC), superior vena cava (SVC) when present, planned extracardiac conduit segment, and branch pulmonary arteries) was segmented semi-automatically with manual refinement. Straight inlet/outlet extensions were added, and measurement planes were positioned away from anastomoses to minimize boundary effects.

For each patient, an initial conduit diameter was planned using the standard geometry-aware approach. Computational fluid dynamics then compared this baseline diameter against adjacent candidates (±2 mm) under identical boundary conditions to quantify energetic cost (PL) and screen for adverse flow features (marked asymmetry or low-velocity regions) prior to final conduit selection.

Meshing was performed in ANSYS ICEM CFD (v19.2) using unstructured tetrahedral elements with prismatic boundary layers (10-12 layers; growth ratio ≤1.2), typically ∼1-2 × 10^6^ elements. Mesh-independence was assessed in a representative subset (*n* = 50); total cavopulmonary PL changed by <3% with refinement. Simulations were performed in ANSYS Fluent (v19.2) assuming steady, incompressible Newtonian flow (ρ = 1060 kg/m³; μ = 0.0035 Pa·s), laminar regime, rigid walls, and no-slip. Convergence required residuals <10^−5^ and global mass imbalance <1%, with stability of PL at convergence.

Total venous inflow was prescribed using a resting cardiac index of 3.0 L/min/m^2^ scaled to body surface area, with an IVC: SVC split of 65:35%. Outlet boundary conditions were 0 D resistance (R-only) outlets, partitioned between branch pulmonary arteries proportional to branch pulmonary artery cross-sectional area (area-weighted resistance split). No RCR/Windkessel compliance element was modelled. Within each patient, inflow and outlet-resistance settings were held constant across candidate diameters.

The primary quantitative metric was total cavopulmonary PL, computed using area-averaged static pressure and flow at predefined planes orthogonal to the local centreline (≥2 vessel diameters from anastomoses):


$$\text{PL}=\Sigma \left(Q\_\text{in}\times \bar{\text{P}}\_\text{in}\right)-\Sigma \left(Q\_\text{out}\times \;\bar{\text{P}}\_\text{out}\right),$$

where Q is volumetric flow rate, and $\bar{\text{P}}$ is area-averaged static pressure at the corresponding plane. Qualitative review of velocity fields/streamlines assessed flow symmetry and low-velocity regions. A change from the baseline planned diameter was recommended when an alternative achieved ≥10% relative PL reduction without introducing adverse qualitative features. If PL differences were <5%, the smaller diameter was favoured to limit oversizing-related low-velocity regions, provided conduit routing feasibility and early intraoperative haemodynamics were acceptable. Final conduit choice remained surgeon-directed and confirmed intraoperatively.

In *n* = 50 cases, inflow was varied by ±15% to assess ranking stability by PL. Where contemporaneous catheter haemodynamics were available (*n* = 120), simulated cavopulmonary pressure gradients were compared descriptively with measured values as a pragmatic face-validity check (not used for calibration).

### Perioperative management and anticoagulation

Perioperative care followed a unified pathway emphasizing low airway pressures, early achievement of negative fluid balance, modified ultrafiltration, and protocolized diuresis, with stepwise escalation for suspected chylothorax to mitigate venous congestion and pleural morbidity.

Systemic corticosteroids were not administered routinely across the study period. At selected centres—predominantly in the later era—an early postoperative methylprednisolone-based protocol was incorporated into the pleural effusion/chylothorax pathway for patients meeting prespecified high-risk criteria, aiming to attenuate inflammatory capillary leak and lymphatic congestion in the immediate post-Fontan phase. For the primary analysis, “corticosteroid therapy” was defined a priori as systemic corticosteroid initiation within 48 hours after EC-TCPC (early-start exposure window). The index agent was intravenous methylprednisolone (methylprednisolone-equivalent dosing), typically 0.5-2.0 mg/kg/day in divided doses for 48-72 hours followed by a short taper per treating team discretion. Indications were protocolized and integrated immediate post-bypass physiology and early drainage profile (eg, elevated CVP, borderline perfusion/cardiac output markers, and/or high early pleural drainage within postoperative days 0-1). Contraindications included suspected/confirmed uncontrolled infection, active gastrointestinal bleeding, or other clinician-determined high-risk conditions; glucose monitoring and stress-ulcer prophylaxis followed intensive care unit (ICU) standards. To minimize reverse causality, steroid initiation beyond the early-start window (late “rescue” therapy after prolonged effusion was established) was not classified as the primary exposure in the main models.

Thromboprophylaxis was initiated with low-molecular-weight heparin and transitioned before discharge to long-term oral anticoagulation per consortium protocol. Warfarin predominated in Era I, whereas from 2018 onward eligible patients increasingly received direct oral anticoagulants (DOACs; predominantly rivaroxaban), with warfarin maintained when DOAC eligibility criteria were not met or contraindications existed. Regimen selection incorporated predefined safety/eligibility considerations (age/weight, hepatic and renal function, bleeding risk, drug interactions, and anticipated adherence) with standardized surveillance across centres, informed by accumulating Fontan thromboprophylaxis evidence including our prior comparative cohort.^14–16^

### Follow-up and outcome adjudication

Patients were evaluated at discharge, at 1, 3, 6, and 12 months, and annually thereafter with clinical assessment, echocardiography, and ECG. Hepatic surveillance included liver biochemistry and ultrasonography, with transient elastography when available and cross-sectional imaging (CT/MRI) pursued when clinically indicated. Criteria for transcatheter fenestration closure were prespecified. Functional assessment included NYHA class and exercise testing when feasible. All adverse events were centrally adjudicated.

### Statistical analysis

Continuous variables are presented as mean (SD) or median (IQR), and categorical variables as *n* (%). Between-group comparisons used Student’s *t*-test or Mann-Whitney *U*-test for continuous variables and χ^2^ or Fisher’s exact test for categorical variables, as appropriate.

Time-to-event outcomes (overall survival and freedom from MAE) were analysed using Kaplan-Meier methods with log-rank tests; patients were censored at last confirmed clinical contact and estimates are reported with 95% confidence intervals (CIs). For non-fatal end-points (thromboembolism and MAE), competing-risk analyses were performed with death as the competing event using cumulative incidence functions, Gray’s test, and multivariable Fine-Gray models (reported as subdistribution hazard ratio [sHR] with 95% CI).

Early outcomes were assessed using multivariable logistic regression; late mortality and other time-dependent outcomes were assessed using Cox proportional hazards models. Centre-level clustering was handled with robust standard errors, and multivariable models included variables with *P* < .10 in univariable analyses and/or established clinical relevance.

To mitigate treatment-selection bias in the late-era CFD comparison, we performed a propensity score overlap-weighting (OW-IPTW) analysis within Era II. The propensity model included age, sex, body surface area, anatomic diagnosis, heterotaxy, preoperative oxygen saturation, mean pulmonary artery pressure, pulmonary vascular resistance, conduit size category, fenestration status, concomitant procedures, centre, and calendar year. Covariate balance was evaluated using standardized mean differences (SMDs), with SMD ≤0.10 indicating acceptable balance. Weighted regression with robust variance estimation was used for early end-points, and weighted Fine-Gray (death as the competing event) and Cox models were used for time-to-event end-points.

Missing outcome data were not imputed. Time-to-event end-points were censored at the last confirmed clinical contact, and vital status ascertainment was used to capture death events where available. Missing covariate data (when applicable) were handled using multiple imputation with chained equations (MICEs) under a missing-at-random assumption, using m = 5 imputed datasets with pooled estimates derived using Rubin’s rules. Variable-level missingness and imputation details are provided in **Tables S3 and S4**.

Sensitivity analyses included parsimonious models and era-restricted analyses.

All statistical analyses were performed using SPSS version 29.0 (IBM Corp., Armonk, NY) and R version 4.3.2 (R Foundation for Statistical Computing, Vienna, Austria). A 2-sided *P* value <.05 was considered statistically significant.

For non-fatal end-points (eg, thromboembolism and MAE), analyses were performed using the available longitudinal clinical follow-up data and were censored at the last confirmed clinical contact; vital status ascertainment was used to capture death events where available.

## Results

### Baseline characteristics

A total of 1171 patients were assessed for eligibility; 383 were excluded because core data required for end-point adjudication were insufficient/unavailable, leaving 788 patients for analysis (**Figure 1**). Mean age at Fontan completion was 7.27 (4.10) years (median 8; range 2-43), 58.1% were male, and mean body surface area was 1.02 (0.21) m^2^. Preoperative oxygen saturation was 81 (6)%, mean pulmonary artery pressure 13.6 (2.8) mmHg, and pulmonary vascular resistance 2.25 (0.60) Wood units.

Primary diagnoses were tricuspid atresia (29.3%), double-inlet left ventricle (23.6%), unbalanced atrioventricular septal defect (20.8%), pulmonary atresia with intact ventricular septum (13.1%), and other variants (13.2%); heterotaxy was present in 11.7% (**Table 2**). Compared with Era I, Era II patients were older with larger body surface area and lower pulmonary vascular resistance, while diagnosis distribution and baseline oxygen saturation were similar (**Table 2**).

**Table 2.** Baseline Characteristics of the Cohort

Characteristic	Overall (*n* = 788)	Era I (2009-2016) (*n* = 392)	Era II (2017-2025) (*n* = 396)	*P* value
Age at Fontan (years), mean (SD)	7.27 (4.10)	6.70 (3.80)	7.80 (4.20)	.002
Male sex, *n* (%)	458 (58.1)	228 (58.2)	230 (58.1)	.980
Body surface area (m²), mean (SD)	1.02 (0.21)	0.98 (0.19)	1.06 (0.22)	.001
Preoperative oxygen saturation (%), mean (SD)	81 (6)	80 (6)	82 (5)	.080
Mean pulmonary artery pressure (mmHg), mean (SD)	13.6 (2.8)	13.8 (2.9)	13.4 (2.7)	.120
Pulmonary vascular resistance (WU), mean (SD)	2.25 (0.60)	2.40 (0.70)	2.10 (0.50)	.010
Anatomic diagnosis, *n* (%)				.450
• Tricuspid atresia	231 (29.3)	112 (28.6)	119 (30.1)	
• Double-inlet left ventricle	186 (23.6)	95 (24.2)	91 (23.0)	
• Unbalanced AVSD	164 (20.8)	83 (21.2)	81 (20.5)	
• PA/IVS	103 (13.1)	51 (13.0)	52 (13.1)	
• Other	104 (13.2)	51 (13.0)	53 (13.4)	
Heterotaxy syndrome, *n* (%)	92 (11.7)	48 (12.2)	44 (11.1)	0.620

Continuous variables are presented as mean (SD) unless otherwise specified; categorical variables are presented as *n* (%). Between-era comparisons were performed using Student’s *t*-test for normally distributed continuous variables or the Mann-Whitney *U*-test for non-normally distributed variables, and the χ^2^ test (or Fisher’s exact test when expected cell counts were <5) for categorical variables. *P* values reflect 2-sided testing.

Abbreviations: AVSD, atrioventricular septal defect; BSA, body surface area; WU, Wood units.

### Operative characteristics

All procedures were performed using expanded polytetrafluoroethylene (ePTFE; Gore-Tex) conduits; ring-reinforced grafts were used selectively based on conduit course and surgeon preference to minimize kinking or extrinsic compression.

Conduit diameters ranged from 12 to 22 mm (including limited use of 12-14 mm conduits in very small/early-era patients), and these were included within the <18 mm conduit category.

Mean conduit diameter was 18.47 (1.90) mm (range 12-22). A small number of patients received 12-14 mm conduits (early-era, very small patients); these cases are included in the <18 mm category.

Fenestration was created in 22.6% (178/788) and was less frequent in Era II than Era I (14.4% vs 30.9%; *P* < .001). Mean cardiopulmonary bypass time was 128 (29) min; among those requiring aortic cross-clamping, mean cross-clamp time was 41 (12) min. Bilateral pulmonary artery mobilization to the hila was achieved in 94.2% overall, increasing modestly in Era II (96.0% vs 92.3%; *P* = .020). Conduit sizing shifted towards larger diameters in Era II (19.1 vs 17.8 mm; *P* < .001) (**Table 1**; **Figure S1**).

### Early postoperative outcomes

Early (≤30-day/in-hospital) mortality was 2.4% (19/788), primarily due to low cardiac output (*n* = 8), refractory arrhythmia (*n* = 4), overwhelming pleural effusion (*n* = 3), or sepsis (*n* = 4). Intensive care unit length of stay was 7.9 days (IQR 5-10) and hospital length of stay was 15.8 (6.5) days.

**Figure 2.**
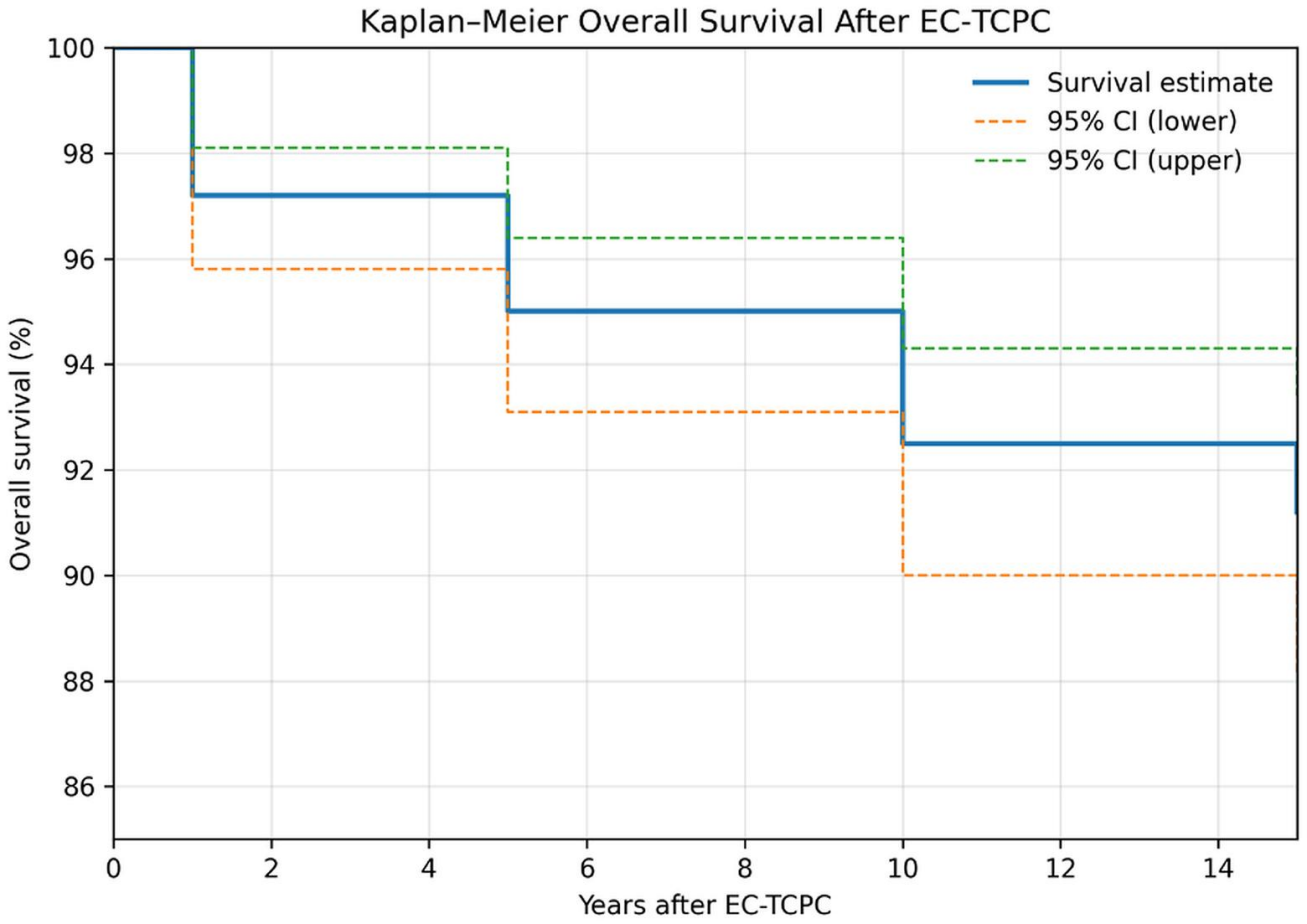
Kaplan-Meier Overall Survival after Extracardiac Total Cavopulmonary Connection (EC-TCPC). Overall survival is shown as the Kaplan-Meier estimate with dashed lines indicating 95% confidence intervals. Time origin was the date of EC-TCPC, with censoring at the last confirmed clinical contact. Abbreviations: CI, confidence interval; EC-TCPC, extracardiac total cavopulmonary connection.

Mean pleural drainage duration was 10.5 (5.2) days and was shorter in Era II (8.4 vs 12.6 days; *P* < .001). Prolonged pleural effusion (>14 days) occurred in 14.1% overall and was less frequent in Era II (9.3% vs 18.7%; *P* < .001). Chylothorax occurred in 4.6% and was managed conservatively with escalation to thoracic duct embolization when required. Reoperation for bleeding occurred in 1.9%. Transient junctional rhythm occurred in 10.4%, and 1.4% required temporary pacing. Era II had shorter ICU stay (7.0 vs 8.5 days; *P* = .020) with similar early mortality and reoperation rates (**Table 3**, **Figure 2**).

**Table 3.** Early Postoperative Outcomes and Complications

Outcome	Overall (*n* = 788)	Era I (*n* = 392)	Era II (*n* = 396)	*P* value
Early mortality, *n* (%)	19 (2.4)	12 (3.1)	7 (1.8)	.180
ICU stay, days—median [IQR]	7.9 [5–10]	8.5 [6–11]	7.0 [4–9]	.020
Hospital stay, days—mean (SD)	15.8 (6.5)	16.5 (6.9)	15.1 (6.0)	.010
Pleural drainage duration, days—mean (SD)	10.5 (5.2)	12.6 (5.3)	8.4 (4.1)	<.001
Prolonged pleural effusion (>14 days), *n* (%)	111 (14.1)	73 (18.7)	38 (9.3)	<.001
Chylothorax, *n* (%)	36 (4.6)	20 (5.1)	16 (4.0)	.420
Reoperation for bleeding, *n* (%)	15 (1.9)	9 (2.3)	6 (1.5)	.370
Transient junctional rhythm, *n* (%)	82 (10.4)	43 (11.0)	39 (9.8)	.550
Temporary pacing required, *n* (%)	11 (1.4)	7 (1.8)	4 (1.0)	.360

Early mortality was defined as death within 30 days of surgery or during the index hospitalization Intensive care unit length of stay is reported as median (IQR); other continuous outcomes are reported as mean (SD) unless otherwise specified. Prolonged pleural effusion was defined as chest tube drainage persisting >14 days. *P* values were obtained using Mann-Whitney *U*-test for ICU stay and Student’s *t*-test for other continuous variables; categorical variables were compared using χ^2^/Fisher’s exact test.

Abbreviations: ICU, intensive care unit; IQR, interquartile range; SD, standard deviation.

### Late outcomes and complications

**Figure 3.**
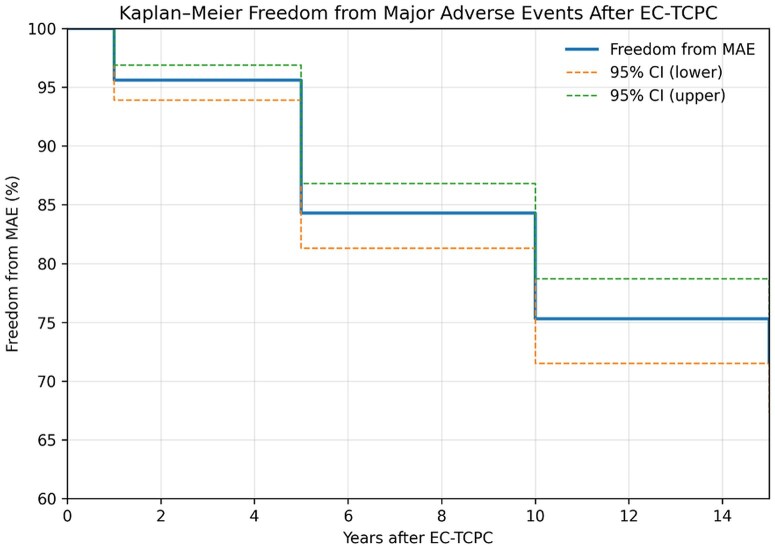
Kaplan-Meier Freedom from Major Adverse Events (MAEs) after EC-TCPC. Major adverse event comprises thromboembolism, protein-losing enteropathy, clinically significant Fontan-associated liver disease, or clinically significant arrhythmia requiring intervention. Dashed lines indicate 95% confidence intervals. Patients were censored at the last confirmed clinical contact. Abbreviations: CI, confidence interval; EC-TCPC, extracardiac total cavopulmonary connection.

Over a mean follow-up of 8.2 (3.9) years (median 8.2 [IQR 4.1-12.5]), late mortality occurred in 4.1% (32/788). Kaplan-Meier overall survival was 97.2% at 1 year, 95.0% at 5 years, and 92.5% at 10 years (**Figure 2**). Late morbidities included arrhythmia requiring intervention (8.9%), thromboembolism (5.2%), PLE (6.2%), clinically significant FALD (9.3%), and plastic bronchitis (0.9%). Reintervention occurred in 7.4% (58/788), most commonly fenestration closure (*n* = 39), branch pulmonary artery intervention (*n* = 12), or conduit revision (*n* = 7). Notably, none of the patients who received 12-14 mm conduits required conduit revision/reoperation during follow-up. Era II had lower PLE (4.1% vs 8.3%; *P* = .020) and FALD (6.8% vs 11.7%; *P* = .040), without significant era differences in arrhythmia or thromboembolism (**Table 4**, **Figure 3**).

**Table 4.** Late Morbidity and Reinterventions

Outcome	Overall (*n* = 788)	Era I (*n* = 392)	Era II (*n* = 396)	*P* value
Late mortality, *n* (%)	32 (4.1)	18 (4.6)	14 (3.5)	.42
Arrhythmia requiring intervention, *n* (%)	70 (8.9)	37 (9.4)	33 (8.3)	.59
Thromboembolic events, *n* (%)	41 (5.2)	23 (5.9)	18 (4.5)	.38
Protein-losing enteropathy, *n* (%)	49 (6.2)	33 (8.3)	16 (4.0)	.02
Fontan-associated liver disease, *n* (%)	73 (9.3)	46 (11.7)	27 (7.1)	.04
Plastic bronchitis, *n* (%)	7 (0.9)	4 (1.0)	3 (0.8)	.72
Any reintervention, *n* (%)	58 (7.4)	32 (8.2)	26 (6.6)	.35
Fenestration closure, *n*	39	22	17	—
PA branch intervention, *n*	12	6	6	—
Conduit revision, *n*	7	4	3	—

Late mortality was defined as death occurring >30 days after surgery. “Arrhythmia requiring intervention” denotes arrhythmia documented on ECG/Holter that required catheter ablation, permanent pacing, or chronic antiarrhythmic therapy. Thromboembolic events required imaging confirmation. Reinterventions include catheter-based and surgical procedures; the subcategories listed (fenestration closure, PA branch intervention, conduit revision) are descriptive counts and are not mutually exclusive if multiple procedures occurred in the same patient over time. *P* values were derived from χ^2^/Fisher’s exact tests, as appropriate.

Abbreviations: FALD, Fontan-associated liver disease; PA, pulmonary artery; PLE, protein-losing enteropathy.

Among late deaths, cause of death could not be ascertained for 14 patients, predominantly cross-border cases residing in different Iraqi cities, due to the absence of a centralized mortality registry and limited access to detailed records.

### Anticoagulation subgroup

All patients received long-term oral anticoagulation: warfarin (*n* = 374) or DOACs (*n* = 414; predominantly rivaroxaban). Thromboembolism was less frequent with DOACs than warfarin (2.6% vs 7.4%; *P* = .008). In competing-risk analysis (death as the competing event), DOAC use was associated with lower thromboembolism risk (sHR 0.52, 95% CI 0.33-0.92; *P* = .033). Major bleeding events were uncommon (8/374 vs 2/414) and permanent treatment discontinuation occurred less frequently with DOACs (17/374 vs 3/414).

### Survival and freedom from events

Kaplan-Meier estimated overall survival was 97.2% (95% CI 95.8-98.1) at 1 year, 95.0% (93.1-96.4) at 5 years, 92.5% (90.0-94.3) at 10 years, and 91.2% (88.1-93.4) at 15 years. Freedom from MAEs was 95.6% (95% CI 93.9-96.9) at 1 year, 84.3% (81.3-86.8) at 5 years, 75.3% (71.5-78.7) at 10 years, and 71.7% (67.3-75.6) at 15 years (**Figure 3**). Freedom from MAE differed by fenestration status (non-fenestrated 90.4% vs fenestrated 81.6%; log-rank *P* = .021); however, fenestrated patients had higher baseline haemodynamic risk and higher immediate post-bypass CVP, consistent with confounding by indication. While overall survival did not differ by conduit size category (≤18 mm vs >18 mm; log-rank *P* = .180), patients with ≤18 mm conduits had higher early postoperative CVP (15.2 vs 13.8 mmHg; *P* = .003) and longer pleural drainage duration (10.4 vs 8.7 days; *P* = .010). Outcomes were benchmarked descriptively against contemporary cohorts and meta-analytic estimates (**Table S1**).

**Figure 4.**
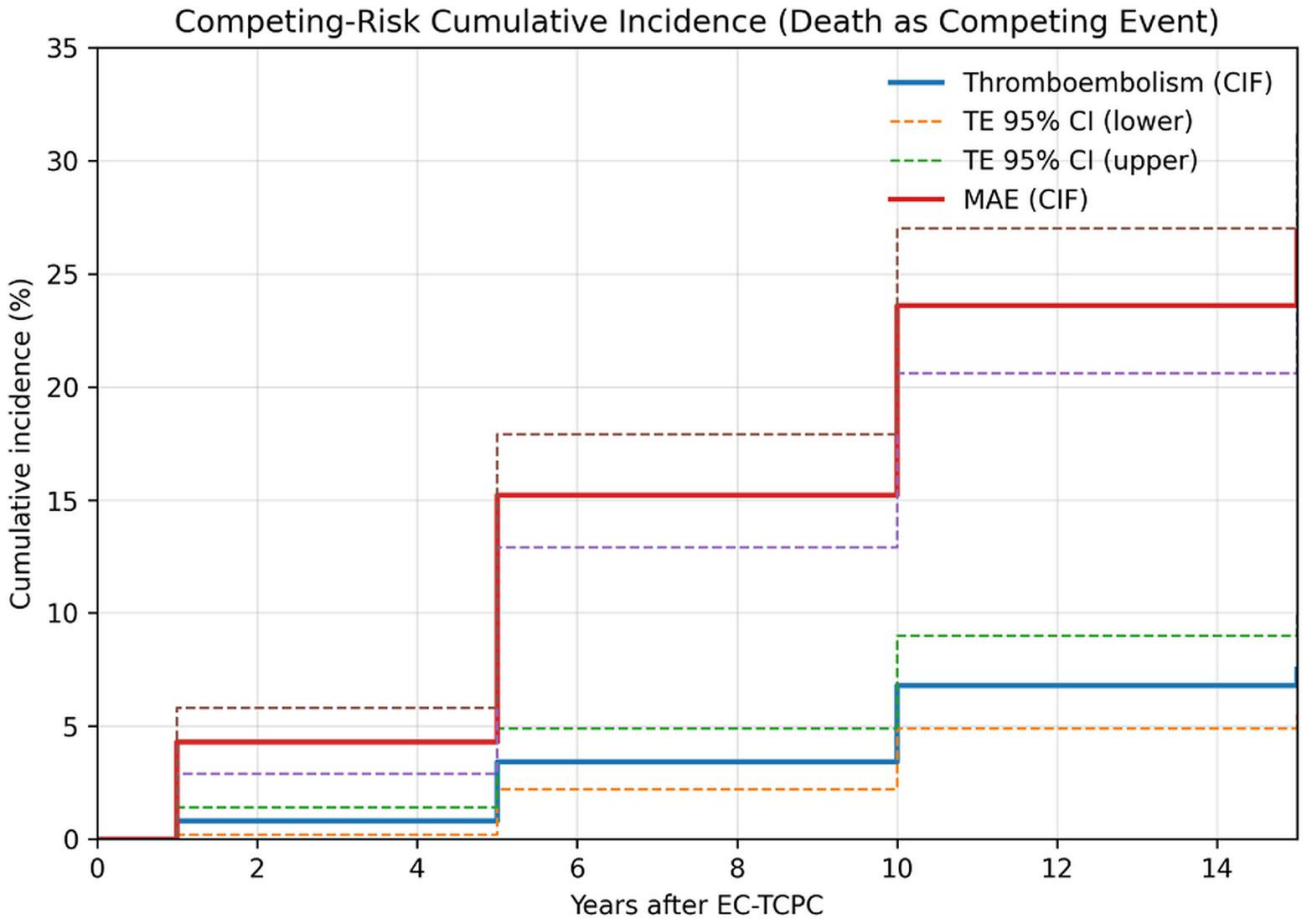
Competing-risk Cumulative Incidence Functions for Thromboembolism and Major Adverse Events (MAEs) after EC-TCPC. Death was treated as the competing event. Curves show cumulative incidence estimates, with dashed lines indicating 95% confidence intervals. Group comparisons (where applicable) were performed using Gray’s test, and subdistribution effects were estimated using Fine-Gray models. Abbreviations: CI, confidence interval; EC-TCPC, extracardiac total cavopulmonary connection; MAE, major adverse event.

In competing-risk analyses with death as the competing event, cumulative incidence of thromboembolism was 0.8% (95% CI 0.2-1.4) at 1 year, 3.4% (2.2-4.9) at 5 years, 6.8% (4.9-9.0) at 10 years, and 7.5% (5.5-10.0) at 15 years. Corresponding cumulative incidence of MAE was 4.3% (2.9-5.8), 15.2% (12.9-17.9), 23.6% (20.6-27.0), and 26.9% (23.4-31.2), respectively (**Figure 4**).

### Multivariable analyses

In multivariable logistic regression, PPE (>14 days) was associated with age <5 years, postoperative CVP >18 mmHg, and absence of early-start systemic corticosteroid therapy (initiated within 48 hours per pathway definition) (aOR 1.90, 95% CI 1.20-3.00; *P* = .008). This estimate reflects an adjusted association in an observational cohort and should not be interpreted as causal. In Cox modelling for late mortality (32 deaths), ICU stay >10 days predicted higher risk (adjusted hazard ratio [aHR] 2.70, 95% CI 1.40-5.30; *P* = .003). In Fine-Gray competing-risk modelling for MAE, heterotaxy was associated with higher MAE risk (sHR 2.00, 95% CI 1.10-3.50; *P* = .008). Centre clustering and sensitivity analyses yielded consistent estimates; full outputs are provided in **Table 5** (**Figure 4**).

**Table 5.** Multivariable Analyses of Key Outcomes

Model/outcome	Predictor	Adjusted effect	95% CI	*P* value	Prespecified adjustment set
A. Prolonged pleural effusion >14 days (Logistic regression; events = 111/788)	Age <5 years (vs ≥5)	aOR 1.80	1.20-2.70	.005	Age at Fontan + sex + era + conduit size category + fenestration + CPB time + heterotaxy + postoperative CVP + centre (cluster-robust SE)
	Postoperative CVP >18 mmHg	aOR 2.30	1.50-3.40	<.001	Same as above
	No corticosteroid therapy	aOR 1.90	1.20-3.00	.008	Same as above
B. Late mortality (Cox proportional hazards; deaths = 32/788)	ICU stay >10 days	aHR 2.70	1.40-5.30	.003	Age at Fontan + sex + era + conduit size category + fenestration + CPB time + heterotaxy + centre (cluster-robust SE)
C. Thromboembolism (Fine-Gray subdistribution hazards; events = 41/788; competing event=death)	DOAC use (vs warfarin)	sHR 0.52	0.33-0.92	.033	Age at Fontan + sex + era + rhythm status + conduit size category + fenestration + heterotaxy + centre (cluster-robust SE)
D. Major adverse events (MAEs) (Fine-Gray subdistribution hazards; events = 158/788; competing event=death)	Heterotaxy (yes vs no)	sHR 2.00	1.10-3.50	.008	Age at Fontan + sex + era + anatomic diagnosis + conduit size category + fenestration + CPB time + postoperative CVP + centre (cluster-robust SE)

Non-fatal late end-points (thromboembolism and MAE) were analysed using cumulative incidence functions with death treated as a competing event; multivariable Fine-Gray models are reported as sHR (95% CI). Time origin was the date of EC-TCPC, with censoring at last confirmed follow-up.

Abbreviations: aHR, adjusted hazard ratio; aOR, adjusted odds ratio; CI, confidence interval; CPB, cardiopulmonary bypass; CVP, central venous pressure; DOAC, direct oral anticoagulant; ICU, intensive care unit; MAE, major adverse events; sHR, subdistribution hazard ratio.

### CFD-guided sizing subcohort (Era II)

In Era II (*n* = 396), CFD-guided conduit sizing was applied in 217 patients, and 179 underwent EC-TCPC without CFD guidance; analyses were adjusted for calendar year and centre effects (cluster-robust standard errors). Computational fluid dynamics recommendations altered the initially planned conduit diameter in 42% of cases (91/217), most commonly upsizing by 2 mm (*n* = 58) or downsizing by 2 mm (*n* = 33). The final implanted conduit sizes in the CFD group were distributed as follows: <18 mm (13.8%), 18-20 mm (69.1%), and >20 mm (17.1%), reflecting integration of CFD outputs with intraoperative haemodynamics and operative judgement.

CFD-guided patients had lower early postoperative CVP (median 14.2 [IQR 13.1-15.6] vs 15.3 [14.0-16.8] mmHg; *P* = .021) and lower PPE (11.9% vs 17.3%; *P* = .048), with a trend towards shorter pleural drainage duration (median 8.6 [6-11] vs 9.8 [7-13] days; *P* = .062). Five-year thromboembolism cumulative incidence was numerically lower but not significant after competing-risk adjustment (sHR 0.72, 95% CI 0.41-1.26; *P* = .24), and 5-year MAE incidence did not differ (Gray *P* = .37) (**Figure S2**). In Era II, propensity score overlap weighting achieved excellent covariate balance between CFD-guided and non-CFD patients (maximum absolute SMD decreased from 1.67 pre-weighting—driven by calendar-year coupling—to 0.02 after weighting; **Table S2**). In the Era II comparison, CFD-guided planning was associated with improved early postoperative haemodynamics and pleural outcomes (early postoperative CVP: 14.2 [IQR 13.1-15.6] vs 15.3 [14.0-16.8] mmHg; *P* = .021; PPE: 11.9% vs 17.3%; *P* = .048; pleural drainage duration: 8.6 [6-11] vs 9.8 [7-13] days; *P* = .062). However, CFD guidance did not demonstrate an independent reduction in mid-term clinical events after competing-risk adjustment: 5-year thromboembolism risk was not significantly different (sHR 0.72, 95% CI 0.41-1.26; *P* = .24), and 5-year MAE incidence also did not differ (Gray *P* = .37). Collectively, these findings support interpreting CFD as a decision-support tool that improves early haemodynamic/pleural recovery, with limited incremental impact on mid-term clinical events in this observational cohort.

## Discussion

In this multicentre cohort of 788 consecutive EC-TCPCs, early mortality was 2.4%, 10-year survival was 92.5%, and freedom from MAEs reached 75.3%. These results are consistent with contemporary extracardiac-dominant series and provide a clinically interpretable benchmark for protocolized Fontan management in high-volume programmes.^4–7^^,^^12^^,^^17^

Era-based comparisons should be interpreted cautiously because follow-up duration is inherently shorter in Era II, and therefore late events in Era II disproportionately reflect early-to-intermediate phases of Fontan trajectory. Accordingly, observed era differences in perioperative end-points (eg, pleural drainage duration, PPE, ICU stay) are best interpreted as signals of pathway refinement rather than evidence of superior long-term durability. Although time-to-event and competing-risk methods partially mitigate differential observation time, residual bias remains, and era should be considered a calendar-time proxy for multiple co-evolving practices rather than an isolated intervention.

Overall survival in our cohort compares favourably with large registry and national datasets reporting 10-year survival in the low 90% range.^4^^,^^5^^,^^7^^,^^18^ Importantly, our study reports adjudicated clinical end-points rather than incidence-rate estimates, facilitating more direct interpretation of complication burden.^5–7^ Rates of arrhythmia, thromboembolism, and PLE were within contemporary ranges, acknowledging the persistent heterogeneity of end-point definitions in Fontan literature.^6^^,^^12^

A geometry-aware approach to conduit sizing represents a central component of our strategy. Experimental and computational studies demonstrate that increasing effective cavopulmonary caliber reduces energy loss within physiological limits, whereas excessive upsizing may promote flow stagnation.^8^^,^^13^ In our cohort, conduits ≤18 mm were associated with higher early CVP and longer pleural drainage. However, patient-specific CFD was implemented only in the most recent subgroup; therefore, these findings support a geometry-aware sizing paradigm rather than a direct causal effect of CFD guidance alone. These conduit-size associations are reported in the Survival and Freedom From Events subsection in the Results section and should be interpreted as early haemodynamic correlates rather than causal determinants of late outcomes.

Interpretation of the CFD-guided subgroup requires particular caution. Computational fluid dynamics-guided planning was restricted to late-era patients and selected anatomies, introducing selection bias and temporal confounding. Although CFD guidance was associated with improved early haemodynamic and pleural outcomes, it did not independently reduce mid-term clinical events after adjustment. Thus, CFD should be regarded as a decision-support tool integrated with surgical judgement rather than a determinant of outcome.

Fenestration-related outcomes are inherently influenced by confounding by indication. Fenestration was selectively applied in patients with higher post-bypass haemodynamic risk. Consequently, the higher event burden observed in fenestrated patients reflects underlying physiological vulnerability rather than a harmful effect of fenestration itself. After multivariable adjustment, fenestration was not independently associated with late mortality, supporting current guideline recommendations favouring individualized, haemodynamically driven fenestration strategies.^17^^,^^19^

Prolonged pleural effusion remains the dominant early morbidity after Fontan completion. In our cohort, younger age, elevated postoperative CVP, and absence of corticosteroid therapy independently predicted prolonged effusion. These findings are consistent with prior reports implicating venous hypertension, lymphatic dysfunction, and inflammatory activation in effusion persistence.^7^^,^^20^ Adjunctive systemic corticosteroids have been reported as a potential option in selected patients with refractory pleural effusions/chylothorax after Fontan completion, although evidence is limited and practice remains heterogeneous.^16^ The temporal reduction in pleural drainage duration supports a pathway-based perioperative management approach. The association between early-start corticosteroid therapy and lower odds of PPE should be interpreted cautiously. Although the exposure was defined within an early postoperative window to reduce reverse causality, residual confounding may persist, including centre/era pathway effects and unmeasured lymphatic vulnerability. Therefore, this signal should be considered hypothesis-generating rather than evidence of a causal treatment effect.

Thromboprophylaxis represents another critical aspect of Fontan care. This era-based transition (from warfarin to DOACs in eligible patients) reflected evolving practice, regulatory availability in paediatric populations, and accumulating evidence supporting DOAC feasibility in Fontan thromboprophylaxis, including our previously published comparative cohort. ^14–16^ In this study, DOACs were associated with fewer thromboembolic events than warfarin without increased major bleeding, consistent with paediatric randomized data and contemporary consensus recommendations.^19^^,^^20^

In this study, DOACs were associated with fewer thromboembolic events than warfarin without increased major bleeding, consistent with paediatric randomized data and contemporary consensus recommendations.^14^^,^^15^^,^^21^ Anticoagulant selection should remain integrated with rhythm surveillance and hepatic assessment, given the progressive nature of FALD.^7^^,^^11^

Late morbidity, particularly FALD and heterotaxy-related adverse events, underscores that durable survival depends on structured, lifelong surveillance rather than isolated procedural success. Emerging consensus definitions provide an important framework for standardized reporting and cross-centre benchmarking.^11^ Within this framework, our findings support an integrated follow-up strategy incorporating hepatic staging, rhythm monitoring, and proactive cavopulmonary pathway optimization.^5^^,^^7^^,^^11^

Overall, this large multicentre extracardiac Fontan experience demonstrates that a physiology-anchored, protocolized strategy yields competitive mid-term survival and acceptable complication rates. While perioperative recovery has improved over time, late Fontan morbidity continues to accumulate, emphasizing the need for continued refinement of individualized surgical planning and long-term surveillance strategies.

From a clinical and methodological standpoint, the novelty of this report is 2-fold. First, we provide benchmark EC-TCPC outcomes with centrally adjudicated complications from a 4-centre consortium spanning 2 health-system contexts, with explicit documentation of cohort assembly and follow-up availability. Second, for non-fatal end-points we prioritized competing-risk reporting to avoid overestimation inherent to Kaplan-Meier methods when death precludes later events. Within this framework, the CFD analysis is intentionally presented as a late-era decision-support marker embedded in co-evolving pathway elements; accordingly, the main message is that CFD-guided planning can be associated with improved early haemodynamic/pleural recovery, while its incremental association with mid-term clinical events is neutral after adjustment and should not be interpreted causally.

Taken together, the manuscript is intended to provide (i) benchmark outcomes for a large real-world EC-TCPC cohort and (ii) hypothesis-generating, risk-adjusted associations for prespecified subgroup comparisons (era, fenestration, conduit size, and late-era CFD decision support), without implying causality.

### Limitations

This study is limited by its retrospective, multicentre design across an era of evolving practice; therefore, causal inferences cannot be made. Although a harmonized consortium pathway was used, temporal changes in perioperative management, fenestration practice, conduit sizing, anticoagulation, and adjunctive therapies may introduce residual confounding despite multivariable adjustment and centre-clustered analyses, and several subgroup findings remain susceptible to confounding by indication. Patient-specific CFD-guided sizing was implemented only in the late-era subcohort and was temporally coupled with broader pathway refinements, limiting isolation of the incremental effect of CFD alone. A proportion of patients (primarily those operated in Iraq) had limited longitudinal follow-up; in this subgroup, cause-of-death could not be reliably determined despite availability of all-cause mortality status in most cases, which may underestimate cause-specific inferences.

Associations involving perioperative adjuncts, including early-start corticosteroid protocols, may be influenced by centre/era practice patterns and residual confounding despite adjustment; therefore, these results should not be interpreted as causal.

Although we performed propensity score overlap-weighted sensitivity analyses for the Era II CFD comparison, residual confounding due to unmeasured factors and temporal pathway coupling cannot be fully excluded; therefore, these associations should not be interpreted as causal.

## Conclusion

In this 4-centre cohort of 788 EC-TCPCs, a physiology-driven, protocolized strategy was associated with low early mortality (2.4%), favourable 10-year survival (92.5%), and acceptable freedom from MAEs (75.3%). Geometry-aware conduit selection, selective fenestration guided by post-bypass haemodynamics, and structured thromboprophylaxis were associated with improved early recovery and competitive mid-term outcomes.

Despite advances in perioperative management, late Fontan morbidity continues to accumulate, underscoring the need for lifelong, risk-stratified surveillance and timely catheter-based optimization. Prospective, multicentre studies using harmonized definitions are required to clarify the incremental contribution of CFD-guided sizing and other pathway components and to further refine strategies aimed at maximizing long-term Fontan durability.

## Supplementary Material

ezag145_Supplementary_Data

## Data Availability

The data underlying this article cannot be shared publicly due to the privacy of individuals that participated in the study. The data will be shared on reasonable request to the corresponding author.
