# Supplementary material for: Outcomes of a Physiology-driven Extracardiac Fontan Strategy Incorporating Computational Fluid Dynamics: A Multicentre Study
Source: Eur J Cardiothorac Surg. 2026 Apr 3;68(4):ezag145. doi: 10.1093/ejcts/ezag145 (PMC13253576; doi:10.1093/ejcts/ezag145)
Supplement: ezag145_Supplementary_Data [file ezag145_supplementary_data.zip › Supplementary_material.docx]

**Table S1. Benchmarking of outcomes after extracardiac Fontan / EC-TCPC against contemporary registries, extracardiac-specific cohorts, and meta-analytic estimates.**

| **Study (year)** | **Setting / Fontan type** | **N** | **Follow-up** | **Early mortality** | **Survival (10y)** | **Survival (20y)** | **Key morbidity benchmarking (as reported)** | **Notes** |
| --- | --- | --- | --- | --- | --- | --- | --- | --- |
| **Present study (2009–2025)** | 4 centers; **EC-TCPC** | 788 | Median 8.2y | 2.4% | 92.5% | NR | TE CIF 10y 6.8%; MAE CIF 10y 23.6%; PLE 6.2%; FALD 9.3%; Arrhythmia 8.9%; Reintervention 7.4% | CIF used for non-fatal endpoints with death as competing event; outcomes not imputed |
| **Korean Fontan Registry (Lee et al., 2024)** | National registry; mixed Fontan types | NR | Long-term registry | NR | 95.7% | 92.1% | Survival 30y 90.2% | Registry includes multiple Fontan modifications; not extracardiac-only. |
| **Matched LT vs EC-TCPC cohort (Kodama et al., 2021)** | Single-center; **LT vs EC-TCPC** | NR | Long-term | NR | 97.9% (overall) | 96.8% (overall) | Freedom from death/HTx/Fontan takedown at 20y: **LT 93% vs EC 82%**; thromboembolism incidence **2.87% patient-years** | Comparative design; several endpoints reported as freedom-from composites and incidence rates. |
| **Extracardiac conduit outcomes meta-analysis (Talla et al., 2025)** | Systematic review/meta-analysis; **extracardiac Fontan only** | 61 studies | ≥3y (study-level) | — | — | — | **Incidence per 100 person-years:**arrhythmia 1.8; thrombotic events 0.2; PLE 0.7; conduit obstruction 0.7; reoperation 1.9; late death 0.3 | Best “extracardiac-only” pooled benchmark; complements KM/CIF reporting. ([PubMed](https://pubmed.ncbi.nlm.nih.gov/39952466/)) |
| **ECC comparative cohort (Yuan et al., 2024; PMC)** | Single-center; **ECC vs dTCPC**(matched) | ECC 268 (matched ECC 40) | Long-term | Similar (P>0.05) | **10y transplantation-free survival (matched ECC): 92.8% ± 5.0** | NR | **10y freedom from Fontan failure (matched ECC): 85.9% ± 6.7** | Gives ECC-specific 10y benchmarks using matched analysis. ([PMC](https://pmc.ncbi.nlm.nih.gov/articles/PMC11845359/)) |

Values are shown **as reported**in the cited sources. Direct numerical comparisons should be interpreted cautiously due to heterogeneity in **case-mix (Fontan type and anatomy), inclusion criteria, follow-up ascertainment, endpoint definitions (e.g., Fontan failure composites vs single events), and statistical reporting**(Kaplan–Meier vs competing-risk cumulative incidence vs incidence rates per person-time). Where a metric was not explicitly reported in the cited source, it is denoted **NR (not reported).** Meta-analytic estimates are presented as **incidence per 100 person-years** when reported, and therefore represent **rate-based**benchmarks rather than time-point survival estimates.

**Table S2. Covariate balance before and after propensity score overlap weighting (OW-IPTW) for the Era II CFD-guided versus non-CFD comparison.**

| **Covariate** | **CFD-guided (unweighted) (n=217)** | **Non-CFD (unweighted) (n=179)** | **SMD (unweighted)*** | **CFD-guided (OW-weighted)†** | **Non-CFD (OW-weighted)†** | **SMD (OW-weighted)*** |
| --- | --- | --- | --- | --- | --- | --- |
| Age at Fontan, years (mean ± SD) | 8.0 ± 4.3 | 7.6 ± 4.1 | 0.09 | 7.8 ± 4.2 | 7.8 ± 4.2 | 0.00 |
| Male sex, n (%) | 130 (60.0) | 100 (56.0) | 0.08 | 58.0% | 58.0% | 0.00 |
| Body surface area, m² (mean ± SD) | 1.08 ± 0.23 | 1.04 ± 0.21 | 0.18 | 1.06 ± 0.22 | 1.06 ± 0.22 | 0.00 |
| Baseline oxygen saturation, % (mean ± SD) | 82 ± 5 | 82 ± 5 | 0.00 | 82 ± 5 | 82 ± 5 | 0.00 |
| Mean pulmonary artery pressure, mmHg (mean ± SD) | 13.2 ± 2.6 | 13.6 ± 2.8 | 0.15 | 13.4 ± 2.7 | 13.4 ± 2.7 | 0.00 |
| Pulmonary vascular resistance, WU (mean ± SD) | 2.05 ± 0.48 | 2.15 ± 0.52 | 0.20 | 2.10 ± 0.50 | 2.10 ± 0.50 | 0.00 |
| Heterotaxy, n (%) | 20 (9.2) | 24 (13.4) | 0.13 | 11.1% | 11.1% | 0.00 |
| Primary diagnosis category, n (%) | TA 70 (32.3)DILV 48 (22.1)Unbal AVSD 43 (19.8)PA/IVS 26 (12.0)Other 30 (13.8) | TA 49 (27.4)DILV 43 (24.0)Unbal AVSD 38 (21.2)PA/IVS 26 (14.5)Other 23 (12.8) | 0.09 | TA 30.1%DILV 23.0%Unbal AVSD 20.5%PA/IVS 13.1%Other 13.4% | TA 30.1%DILV 23.0%Unbal AVSD 20.5%PA/IVS 13.1%Other 13.4% | 0.00 |
| Conduit size category, n (%) | <18 mm 30 (13.8)18–20 mm 150 (69.1)>20 mm 37 (17.1) | <18 mm 40 (22.3)18–20 mm 108 (60.3)>20 mm 31 (17.3) | 0.19 | <18 mm 17.7%18–20 mm 65.2%>20 mm 17.2% | <18 mm 17.7%18–20 mm 65.2%>20 mm 17.2% | 0.00 |
| Fenestration, n (%) | 25 (11.5) | 32 (17.9) | 0.18 | 14.4% | 14.4% | 0.00 |
| Any concomitant procedure, n (%) | 60 (27.6) | 66 (36.9) | 0.20 | 31.8% | 31.8% | 0.00 |
| Center (categorical)‡ | Categorical distribution | Categorical distribution | 0.15 | Balanced | Balanced | 0.02 |
| Calendar year (mean ± SD) | 2022 ± 2.0 | 2019 ± 1.5 | 1.67 | 2021 ± 2.5 | 2021 ± 2.5 | 0.00 |

**Table S2. Covariate balance before and after propensity score overlap weighting (OW-IPTW) for the Era II CFD-guided versus non-CFD comparison.** Unweighted columns report raw group summaries (CFD-guided, n=217; non-CFD, n=179). OW-weighted columns report overlap-weighted means/proportions (not counts), reflecting the pseudo-population emphasizing patients in the region of common support. Standardized mean differences (SMDs) are presented as **absolute values**, with **SMD <0.10** indicating adequate covariate balance. Center is a multi-level categorical variable; its balance is summarized using the **maximum absolute SMD across indicator levels.** Calendar year was included to account for temporal coupling of CFD adoption and contemporaneous pathway refinements.

**Abbreviations:** AVSD, atrioventricular septal defect; BSA, body surface area; CFD, computational fluid dynamics; DILV, double-inlet left ventricle; mPAP, mean pulmonary artery pressure; OW-IPTW, overlap-weighted inverse probability of treatment weighting; PA/IVS, pulmonary atresia with intact ventricular septum; SD, standard deviation; SMD, standardized mean difference; TA, tricuspid atresia; WU, Wood units.

**Table S3. Availability of longitudinal outcomes and follow-up completeness**

| **Outcome / variable** | **Complete adjudication available, n (%)** | **Limited/partial follow-up, n (%)** | **Reason** | **Notes** |
| --- | --- | --- | --- | --- |
| Major adverse events (MAE) adjudication | 436 (55.3%) | 352 (44.7%) | Cross-border follow-up constraints (predominantly cases operated in Iraq) | MAE comprises thromboembolism, PLE, clinically significant FALD, or clinically significant arrhythmia requiring intervention. |
| Thromboembolism ascertainment | 436 (55.3%) | 352 (44.7%) | Cross-border follow-up constraints (predominantly cases operated in Iraq) | Imaging-confirmed events. |
| Protein-losing enteropathy (PLE) ascertainment | 436 (55.3%) | 352 (44.7%) | Cross-border follow-up constraints (predominantly cases operated in Iraq) | — |
| Fontan-associated liver disease (FALD) ascertainment | 436 (55.3%) | 352 (44.7%) | Cross-border follow-up constraints (predominantly cases operated in Iraq) | Clinically significant FALD per site surveillance. |
| Arrhythmia requiring intervention ascertainment | 436 (55.3%) | 352 (44.7%) | Cross-border follow-up constraints (predominantly cases operated in Iraq) | Ablation/pacing/chronic antiarrhythmic therapy. |
| Reintervention ascertainment | 436 (55.3%) | 352 (44.7%) | Cross-border follow-up constraints (predominantly cases operated in Iraq) | Includes fenestration closure, PA intervention, and conduit revision. |
| Vital status availability | 733 (93.0%) | 55 (7.0%) unavailable | Registry limitations and geographic dispersion in limited follow-up subgroup | 733 = 436 complete adjudication + 297/352 with vital status available in the limited/partial subgroup. |
| Cause of death (among 51 deaths: 19 early + 32 late) | 37 (72.5%) | 14 (27.5%) unascertained | Absence of a unified mortality registry and cross-border dispersion | All 14 unascertained causes occurred among late deaths in the limited/partial subgroup; early in-hospital deaths were fully ascertained. |

**Table S3.** Availability of longitudinal outcomes and follow-up completeness. “Complete adjudication available” denotes patients with longitudinal clinical follow-up sufficient for prespecified non-fatal outcome ascertainment (n=436). “Limited/partial follow-up” denotes cross-border follow-up constraints in which comprehensive longitudinal adjudication was not consistently available (n=352; predominantly cases operated in Iraq). Within the limited/partial subgroup (n=352), partial longitudinal clinical contact for non-fatal outcome ascertainment was available in 289 and unavailable in 63. For time-to-event analyses, patients were censored at the last confirmed clinical contact; outcomes were not imputed.

**Abbreviations:** FALD, Fontan-associated liver disease; MAE, major adverse events; PA, pulmonary artery; PLE, protein-losing enteropathy.

**Table S4. Missing data in key baseline and operative covariates and imputation details (paste-ready)**

| **Variable** | **Missing n (%)** | **Handling** | **Notes** |
| --- | --- | --- | --- |
| Age at Fontan | 0 (0.0) | Not applicable | Fully available |
| Sex | 0 (0.0) | Not applicable | Fully available |
| Body surface area (BSA) | 5 (0.6) | MICE (covariate imputation) | Imputed using age/anthropometrics and related covariates |
| Preoperative oxygen saturation (%) | 20 (2.5) | MICE (covariate imputation) | Imputed using preoperative haemodynamics and diagnosis |
| Mean pulmonary artery pressure (mPAP, mmHg) | 40 (5.1) | MICE (covariate imputation) | Imputed using PVR, oxygen saturation, and other haemodynamics |
| Pulmonary vascular resistance (PVR, WU) | 50 (6.3) | MICE (covariate imputation) | Imputed using mPAP and related variables |
| Anatomic diagnosis | 0 (0.0) | Not applicable | Fully available |
| Heterotaxy syndrome | 0 (0.0) | Not applicable | Fully available |
| Conduit diameter | 0 (0.0) | Not applicable | Fully available |
| Fenestration status | 0 (0.0) | Not applicable | Fully available |
| Cardiopulmonary bypass time | 15 (1.9) | MICE (covariate imputation) | Imputed using operative complexity and era |
| Postoperative central venous pressure (CVP) | 30 (3.8) | MICE (covariate imputation) | Imputed using early postoperative haemodynamics and fenestration status |
| Pleural drainage duration (days) | 25 (3.2) | Not imputed (available-case) | Early postoperative outcome; not imputed per outcome-missingness policy |

**Footnote:** Multiple imputation with chained equations (MICE) was applied to missing **covariates** (not outcomes) under a missing-at-random assumption using **m = 5** imputed datasets, with pooled estimates derived using Rubin’s rules.

**Abbreviations:** BSA, body surface area; CVP, central venous pressure; MICE, multiple imputation with chained equations; mPAP, mean pulmonary artery pressure; PVR, pulmonary vascular resistance; WU, Wood units.

**Table S5. Center contributions to the analytic cohort and follow-up ascertainment.**

| **Centre** | **Included in analysis, n** | **Complete adjudication available for prespecified non-fatal outcomes, n** | **Limited/partial follow-up, n** | **Partial longitudinal clinical contact within limited/partial group, n** | **Vital status available, n** |
| --- | --- | --- | --- | --- | --- |
| Çukurova University Faculty of Medicine (Adana, Turkey) | 47 | 47 | 0 | — | 47 |
| Medicana International Beylikdüzü Hospital (Istanbul, Turkey) | 150 | 150 | 0 | — | 150 |
| Istanbul Emsey Hospital and Beykent University Hospital (Istanbul, Turkey) | 239 | 239 | 0 | — | 239 |
| Fallujah Teaching Hospital (Iraq) | 352 | 0 | 352 | 289 | 297 |
| **Total** | **788** | **436** | **352** | **289** | **733** |

**Table S5.** Center-level distribution of the analytic cohort and follow-up ascertainment. Complete adjudication available for prespecified non-fatal outcomes” denotes patients with longitudinal data sufficient for adjudication of prespecified non-fatal endpoints (e.g., thromboembolism, PLE, clinically significant FALD, arrhythmia requiring intervention, and reintervention). “Limited/partial follow-up” denotes cross-border follow-up constraints in which comprehensive longitudinal adjudication was not consistently available (predominantly cases operated in Iraq). Within the limited/partial follow-up group (n=352), partial longitudinal clinical contact for non-fatal outcome ascertainment was available in 289 and unavailable in 63. Vital status could be ascertained for 297/352 patients in the limited/partial group. “—” indicates not applicable.
**Abbreviations:** FALD, Fontan-associated liver disease; PLE, protein-losing enteropathy.
