# Supplementary figures and images for: Outcomes of a Physiology-driven Extracardiac Fontan Strategy Incorporating Computational Fluid Dynamics: A Multicentre Study
Source: Eur J Cardiothorac Surg. 2026 Apr 3;68(4):ezag145. doi: 10.1093/ejcts/ezag145 (PMC13253576; doi:10.1093/ejcts/ezag145)

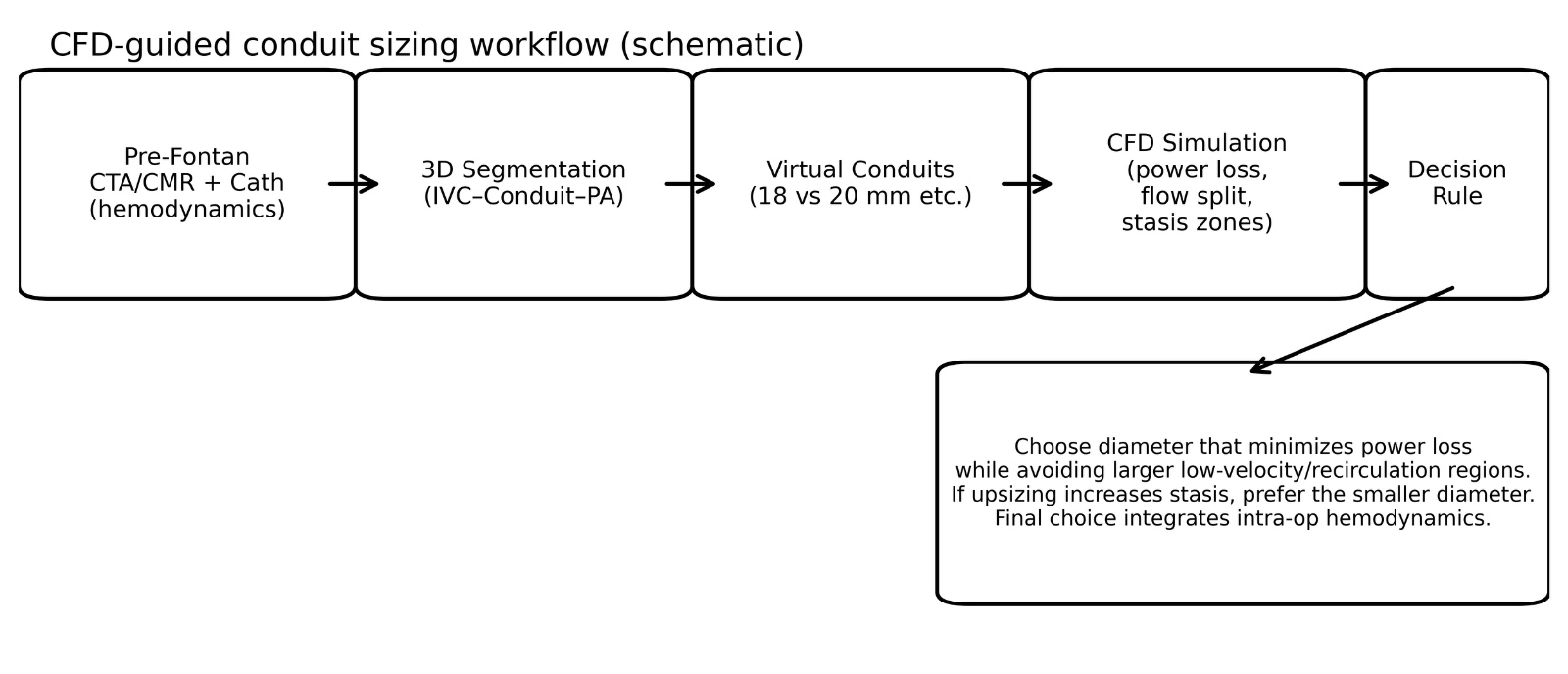

Supplement: ezag145_Supplementary_Data [file ezag145_supplementary_data.zip › FigureS2_600dpi.tif]

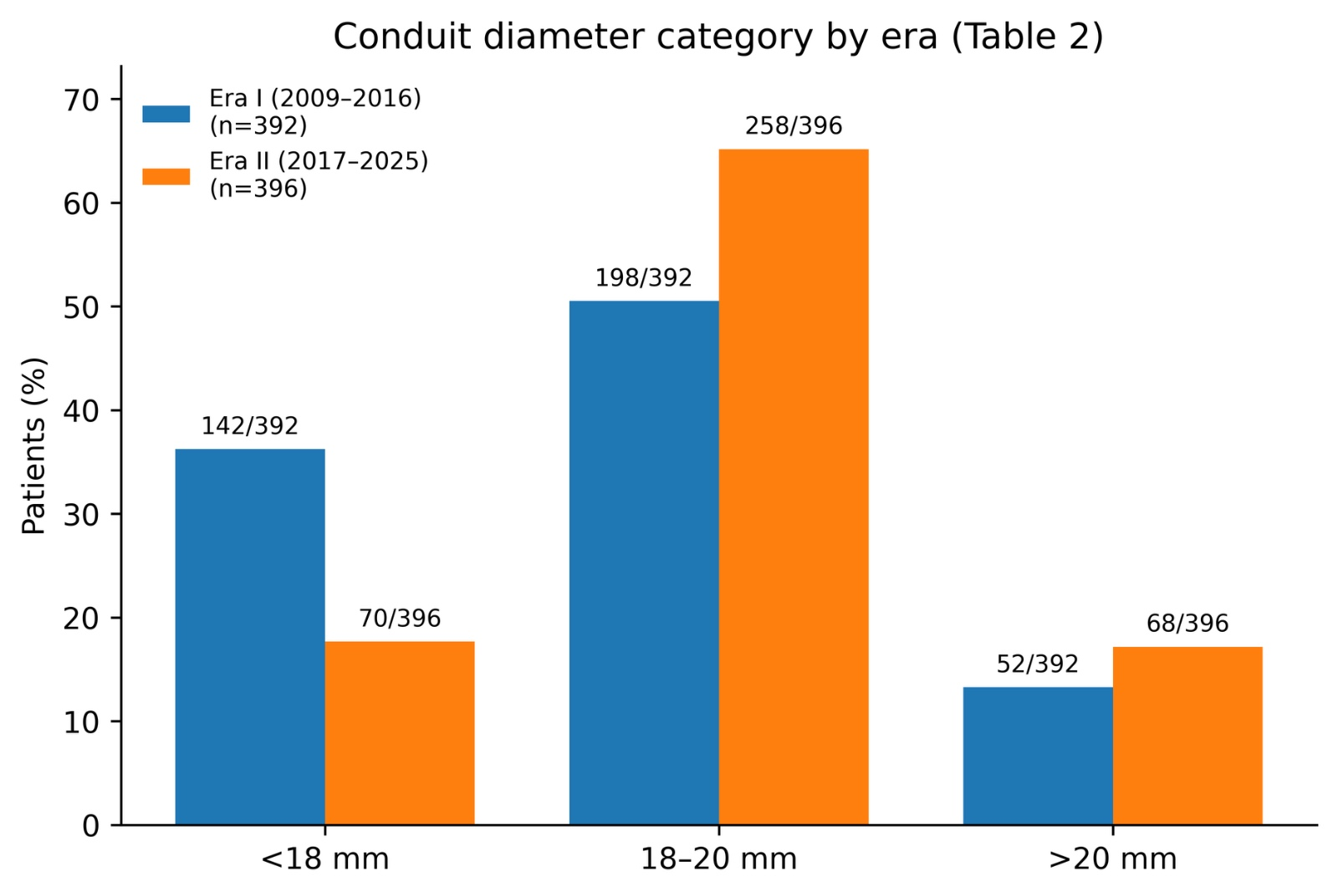

Supplement: ezag145_Supplementary_Data [file ezag145_supplementary_data.zip › FigureS1_600dpi.tif]
